# Capture and Detection of Aerosolized Fentanyl in a Suspended Electrochemical Cell

**DOI:** 10.1021/acs.analchem.4c01321

**Published:** 2024-06-19

**Authors:** Kathryn J. Vannoy, Lynn E. Krushinski, Jeffrey E. Dick

**Affiliations:** † Department of Chemistry, Purdue University, West Lafayette, Indiana 47907, United States; ‡ Elmore Family School of Electrical and Computer Engineering, Purdue University, West Lafayette, Indiana 47907, United States

## Abstract

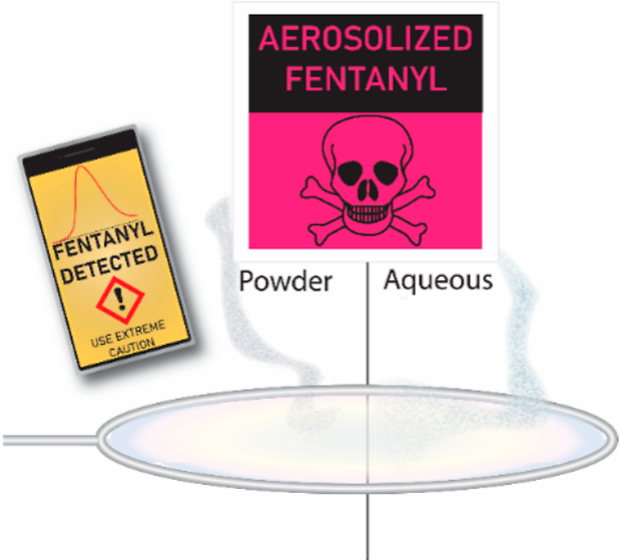

Fentanyl is an extremely potent opioid that is commonly laced into other drugs. Fentanyl poses a danger to users but also to responders or bystanders who may unknowingly ingest a lethal dose (∼2 mg) of fentanyl from aerosolized powder or vapor. Electrochemistry offers a small, simple, and affordable platform for the direct detection of illicit substances; however, it is largely limited to solution-phase measurements. Here, we demonstrate the hands-free capture and electroanalyzation of aerosols containing fentanyl. A novel electrochemical cell is constructed by a microwire (cylindrical working electrode) traversing an ionic liquid film that is suspended within a conductive loop (reference/counter electrode). We provide a quantitative finite element simulation of the resulting electrochemical system. The suspended film maintains a high-surface area:volume, allowing the electrochemical cell to act as an effective aerosol collector. The low vapor pressure (negligible evaporation) of ionic liquid makes it a robust candidate for in-field applications, and the use of a hydrophobic ionic liquid allows for the extraction of fentanyl from solids and sprayed aqueous aerosols.

## Introduction

The opioid crisis is characterized not by a drastic increase of opioid users, but rather an alarming increase in overdose deaths, reaching unprecedented levels during the COVID-19 pandemic.^1^ In the United States, there were more than 80,000 opioid overdose deaths in 2021, and ∼70,000 of those deaths were attributed to synthetic opioids (primarily fentanyls).^2^ The ingestion of small amounts of fentanyl can cause death. The adulteration of heroin and other substances with fentanyl continues to threaten the lives of unknowing users, first responders, and bystanders.^1^

To help combat the crisis, in-field forensic detection strategies have been given a lot of attention. Powerful analytical instruments have been adapted for field work;^3^ for example, the TruNarc Raman spectrometer (ThermoFisher Scientific; Waltham, MA) was recently commercialized. These instruments are highly discriminatory and can identify substances without sampling; however, they cost tens of thousands of dollars and require scientific training to operate, interpret, and maintain.^3^ As such, developing simpler and more affordable techniques remains valuable.^4^ Lateral flow immunoassay fentanyl test strips operating on an antibody detection scheme have recently become available.^5^ For this type of detection strategy, the suspected fentanyl must be sampled and dissolved into a solution in ≥ μM concentrations.^5^ Electrochemical detections are also being explored for in-field fentanyl sensors due to the small, affordable, fast, and simple nature of the instrumentation and methods. Wang’s group has developed modified-glove electrochemical sensors for easy sampling and rapid identification of illicit drugs.^6^ The thumb is used to collect a powder sample, and the index finger contains a flexible sensor chip for the sensing. The sensor chip is coated with a hydrogel film (modified with an ionic liquid/carbon nanotube composite) that allows for electrochemical connection between the electrodes.^7^ Fentanyl can be directly oxidized to give an identifiable peak, allowing for the rapid analysis of unknown powders by relatively controlled sampling and solubilization of the powder.

Most affordable sensors for fentanyl require the sampling of a suspicious powder. This is a dangerous requirement as small amounts of aerosolized fentanyls powder can cause death.^8,9^ Recent overdose trends indicate that deaths from smoking increased by nearly 75% (from 2020 to 2022) and is largely associated with an increase in the smoking of fentanyls with and without commonly smoked stimulants.^10^ As a result, smoking became the predominant route of use associated with overdose deaths in the Midwest and West. Thus, a fentanyl sensor that reports on the fentanyl in the air, in both solid and liquid (e.g., vapors) aerosols, is needed for the safety of those in the field.

Previously, our laboratory designed a variety of analytical methods for the electroanalysis of aerosol contents using particle-into-liquid sampling.^11,12^ In 2022, we presented a soap bubble wall electrochemical cell for the capture and analysis of liquid aerosols containing methamphetamine.^13^ The soap bubble wall was made from 0.1 M KCl and 0.1% Triton X-100, and a microwire working electrode and a platinum wire (1 mm diameter) counter/reference electrode were pushed through the bubble wall. Methamphetamine was detected by direct oxidation at the carbon fiber working electrode. While this study showed that liquid aerosol collection and electrochemical detection is possible in a soap bubble wall, evaporation caused the bubble wall to “pop” every 10 s of seconds, severely limiting the applicability.

Here, we present a novel electrochemical cell for the capture and analysis of solid and liquid fentanyl aerosols. A carbon fiber microwire [*d* = 7 μm] (working electrode) traverses an ionic liquid film that is suspended in a platinum wire loop (counter/reference electrode). The negligible vapor pressure of the ionic liquid not only allows for a robust, long-standing film but also offers the potential for quantitation of electroactive species if one knows the geometry. We present a finite elements model of the unique electrochemical system and show that the experimental results match closely with the simulation. Finally, we demonstrate the use of a suspended hydrophobic ionic liquid for the collection of aerosolized fentanyl. We show that the measured current around 1.2 V versus platinum wire can be diagnostic of fentanyl exposure, where the fentanyl was introduced into the suspended film as loose powder or from liquid aerosols by liquid/liquid extraction. We believe that this detection strategy could be useful for first responders that collect unknown substances, those who provide aid to overdose victims, and people who otherwise come into contact with fentanyl.

## Materials and Methods

### Chemicals and Materials

Ferrocenemethanol (97%), 1-butyl-3-methylimidazolium hexafluorophosphate (for catalysis, ≥98.5%), fentanyl (USP reference standard), and platinum wire (0.5 mm diameter, 99.9% metals basis) were purchased from Sigma-Aldrich. Millipore water (Millipore Milli-Q, 18.20 MΩ·cm^–1^) was used to prepare the aqueous solutions. The P-55 pitch-based carbon fibers (*d* = 7 μm) were obtained from Thornell.

### Electrochemical Cell Construction and Characterization

**Figure 1:**
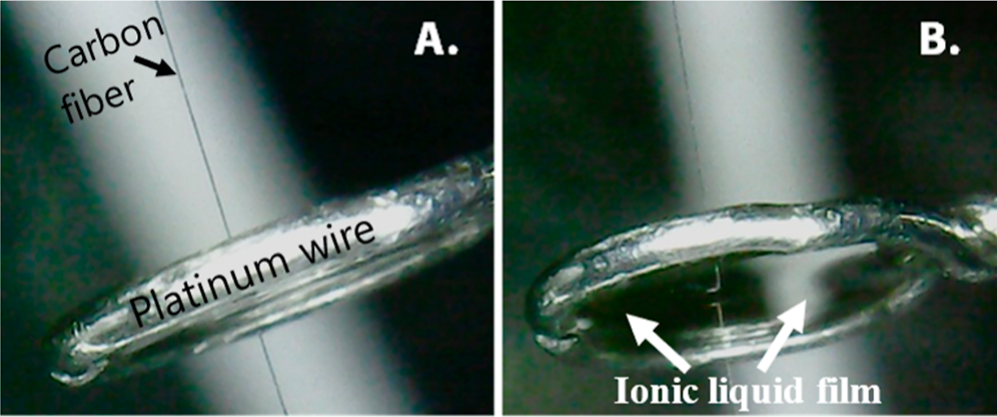
iPhone 12 photographs of the electrochemical cell: a platinum wire (*d* = 0.5 mm) loop containing the suspended ionic liquid film and the carbon fiber (*d* = 7 μm) traversing the film. (A) Side view with the electrodes labeled (B) angled view with the ionic liquid film labeled.

To create the working electrode, a carbon fiber (*d* = 7 μm) was secured onto a support made from a stripped electrical wire with gallium (stored at 55 °C to maintain a liquid state) and hot glue. The carbon fiber and wire support were held in place with a clamp and were connected to the working electrode lead of a CHI 6284E potentiostat. To create the ionic liquid film support, one end of a platinum wire (*d* = 0.5 mm) was wrapped around a circular support. On the other end on the wire, copper tape was wrapped around the straight portion of the platinum support such that a strong electrical connection could be made between the worn loop and the potentiostat leads. A small slit was cut into the platinum wire loop such that the carbon fiber working electrode could be passed through. The suspended ionic liquid film was created by dipping the platinum wire support into a small vial containing the ionic liquid. The wire support was then attached to a Sutter MPC-200 micropositioner and connected to the counter and reference leads of the potentiostat. Images of the described experimental setup can be seen in Figure 1, and a cross-sectional scheme of the resulting electrochemical cell is given in Figure S3.

After the electrochemical cell was created, a solution of 3.55 mM ferrocenemethanol in 1-butyl-3-methylimidazolium hexafluorophosphate was prepared gravimetrically (using the reported density of 1-butyl-3-methylimidazolium hexafluorophosphate [1.38 g·mL^–1^]). The solution was sonicated for 15 min such that the ferrocenemethanol was fully dissolved. Cyclic voltammetry was then performed in the bulk ferrocenemethanol solution with a CHI Pt ultramicroelectrode (*r* = 5 μm) as the working electrode and a Pt wire (*d* = 0.5 mm) as the reference/counter electrode. Voltammetry was performed from −0.1 to 0.4 V at a scan rate of 1 mV·s^–1^. After the bulk measurement, the ferrocenemethanol solution was used to create a suspended film (in the manner described above) in the Pt wire support. The carbon fiber working electrode was then placed into the center of the film, and voltammetry was performed with the Pt wire support as the reference/counter electrode.

### Voltammetry of Fentanyl in Ionic Liquid

All sample preparations and experiments involving fentanyl were performed in a fume hood as fentanyl is acutely toxic. A 3.5 mM fentanyl solution was prepared gravimetrically in 1-butyl-3-methylimidazolium hexafluorophosphate. The solution was sonicated for 1 h to ensure that all fentanyl was dissolved. After the solution was prepared, voltammetry was taken in the bulk with a glassy carbon electrode (CHI, *d* = 3 mm) working electrode. A two-electrode cell was used with a platinum wire counter/reference electrode, where linear sweep voltammetry was taken from 0 to 2 V at 50 mV·s^–1^. Afterward, the 3.5 mM fentanyl solution was used to prepare a suspended film within the Pt wire support. Linear sweep voltammetry was then performed within the film with the Pt wire support as the counter/reference electrode and a carbon fiber as the working electrode from 0 to 1.5 V at a scan rate of 50 mV·s^–1^. Unless otherwise stated, each voltammogram was collected in a freshly constructed ionic liquid film. Between measurements, the platinum wire support was rinsed with ethanol and cleaned with a Kimwipe until no ionic liquid remained visible. The carbon fiber was cleaned with the same procedure or replaced (these fibers sometimes broke or lost connection when removed from the hole in the platinum wire).

### Solid and Liquid Aerosol Sampling and Fentanyl Detection

For both solid and liquid aerosol sampling, a suspended film of the ionic liquid (with no added fentanyl) was threaded with a carbon fiber working electrode, and a background voltammogram was taken. For solid sampling, a small amount of fentanyl powder (<1 mg) was pressed into a glass capillary. The capillary was then carefully touched to the ionic liquid film. After solid sampling, the carbon fiber working electrode was moved the site of the sampling (which could be visualized by powder accumulation) before voltammetry was taken. For aerosol sampling, a solution of 0.5 mM fentanyl was prepared in Millipore water. The solution was sonicated for 30 min and was filtered with a 2.5 μm filter prior to nebulization. It should be noted that significant amounts of fentanyl remained undissolved after sonication, such that filtering was necessary. We estimate the final solution to be on the order of ∼100 μM. All solutions were nebulized with a Aeroneb Solo Nebulizer connected to a Aeroneb Pro-X controller purchased from Aerogen. Solutions were sprayed for 30 s, 1 min, and then 2 min (for a total spray time of 3 min and 30 s).

## Results and Discussion

A redox species with fast one-electron-transfer kinetics was used to characterize the electrochemical system. Figure S1 shows representative cyclic voltammetry of 3.55 mM ferrocenemethanol in a bulk solution of 1-butyl-3-methylimidazolium hexafluorophosphate (ionic liquid). From the limiting anodic current, the diffusion coefficient of ferrocenemethanol in 1-butyl-3-methylimidazolium hexafluorophosphate was determined to be (3.5 ± 0.1) × 10^–12^ m^2^·s^–1^. This value is similar to previously reported values (∼1 × 10^–12^ m^2^·s^–1^).^14^

A platinum loop was then dipped into the ionic liquid solution, forming a suspended liquid film. The loop acts as both the support for the ionic liquid and the counter/reference electrode. We chose platinum as the material because it is malleable and resistant to corrosion but other conductive wires could be used. Ionic liquid was suspended in the wire loop, and a microwire was inserted into the suspended film through a small break in the wire loop. Microwire entry through the side of the loop avoids excessive ionic liquid contamination/wetting that can occur if the wire were to be pushed through film. In this case, we used a carbon fiber (*d* = 7 μm) microwire because fentanyl is directly oxidizable on carbon surfaces within reasonable potential windows (Figure S2). Figure 1 shows photographs of the 1-butyl-3-methylimidazolium hexafluorophosphate solution suspended in a loop (4 mm outer diameter) constructed from platinum wire (*d* = 0.5 mm). The electrode materials, ionic liquid, and conductive loop dimensions are all tunable parameters that may allow this type of electrochemical cell to be useful for a variety of detection modalities and analytes, which will be explored in future directions.

The effective working electrode is a baseless cylinder, or an “annular band” geometry,^15^ and a cross-sectional scheme is given in Figure S3. Scan rate analysis (where voltammetry was performed in the suspended ionic liquid film containing 3.55 mM ferrocenemethanol at various scan rates) indicates that the flux is diffusion controlled (Figure S4) and behaves as expected in this geometry.^16^ Previously Aoki^16^ and Compton^15^ described current at microwires. For thick films, where the length of the baseless cylinder is ∼10^4^× larger than the radius of the microwire, it is reasonable to estimate the current assuming flux to an infinitely long cylinder (neglecting the ends).^15^ As we are using a relatively thin film, numerical simulations were used to understand the current response and extract the effective electrode length.^15^

**Figure 2:**
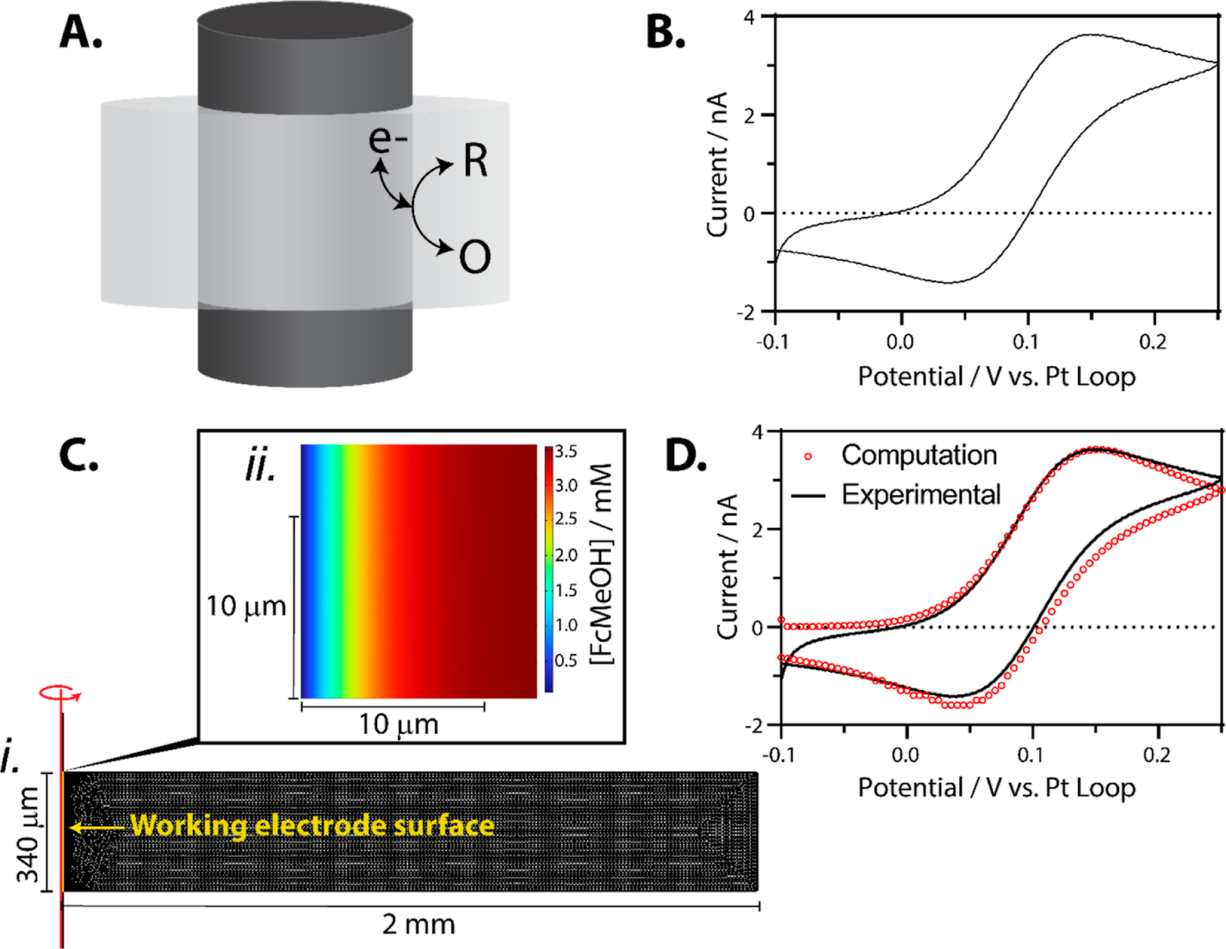
(A) Scheme of the electrochemistry occurring at the interface between the ionic liquid suspended film and the carbon fiber microwire. (B) Representative cyclic voltammogram of 3.55 mM ferrocenemethanol in the ionic liquid suspended film. A two-electrode setup was used with a platinum wire loop containing the suspended ionic liquid acting as the counter/reference electrode and a carbon fiber traversing the film (*d* = 7 μm) acting as the working electrode. The scan starts at −0.1 V, sweeps to 0.25 V, and then back to −0.1 V at a scan rate of 50 mV·s^–1^. (C) (i) 2D-axisymmetric geometry for the COMSOL simulation where the red line (*r* = 0) indicates the axis of symmetry. The surface of the working electrode is shown at *r* = 3.5 μm. The suspended ionic liquid film is represented by the gray rectangle (covered in black mesh lines), and the height of this rectangle was the only adjustable parameter in the simulation (shown here as 340 μm). (ii) *A* ∼ 10 × 10 μm view of the diffusion layer is given where the color map indicates the concentration of ferrocenemethanol at *t* = 7 s into the voltammetry (*E* = 0.25 V). (D) Overlay of computational and experimental voltammetry, where the only adjustable parameter was the height of the film. For this fit, the height of the film was set to 340 μm. In line with the IUPAC convention, anodic current is represented as positive for all panels in this figure.

Figure 2A illustrates cyclic voltammetry occurring at the interface of the cylindrical microelectrode and the suspended ionic liquid film, and a cyclic voltammogram of 3.55 mM ferrocenemethanol is given in Figure 2B. The slight peaking/quasi steady-state voltammetric shape is a result of mass transfer to the electrode.^15−17^ In Figure 2C, we show the 2D-axisymmetric geometry used for the finite element model. For the computation, we assumed that wetting along the microwire was negligible (minimized by threading the working electrode as described above), and the film thickness is homogeneous; thus, the thickness at any point can be described by the effective electrode length. We calculated the film thickness/electrode length by fitting computed data to experimental voltammetry, where the height of the ionic liquid film (Figure 2C, gray box with black meshing) was the only adjustable parameter. Fitting to the voltammogram given in Figure 2B, the ionic liquid/microwire interface/film thickness was calculated to be 340 μm. Details of the computation are given in the Supporting Information (Figures S5–S6). Figure 2D shows the close fit between the experimental voltammetry and simulated voltammetry, suggesting that the method is capable of analyte concentration quantitation if the length of the electrode/ionic liquid interface is fixed. With current fabrication methods, the film thickness varies significantly from sensor to sensor, resulting in a variable current (Figure S7). Future directions will aim to develop quantitative sensors, where the film thickness is standardized (e.g., with surfactants) or the current is normalized. Here, we present a qualitative sensor for the detection of fentanyl in aerosols, an application that benefits from a binary result.

**Figure 3:**
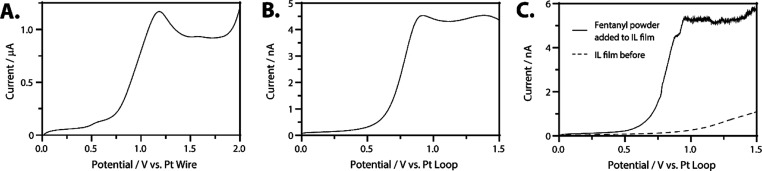
(A) Voltammetry of the 3.5 mM fentanyl in the ionic liquid (1-butyl-3-methylimidazolium hexafluorophosphate). A two-electrode cell was used with a platinum wire counter/reference electrode and a CHI glassy carbon disk (*d* = 3 mm) working electrode. The scan starts at 0 V and sweeps to 2 V at 50 mV·s^–1^. (B) Voltammetry of the ionic liquid suspended film containing 3.5 mM fentanyl. Two-electrodes were used with a platinum wire loop containing the suspended ionic liquid acting as the counter/reference electrode and a carbon fiber (*d* = 7 μm) traversing the film acting as the working electrode. The scan starts at 0 V and sweeps to 1.5 V at 50 mV·s^–1^. (C) Overlaid voltammetry in the ionic liquid suspended film before (dashed) and after (solid) the addition of fentanyl powder. Two-electrodes were used with a platinum wire loop containing the suspended ionic liquid acting as the counter/reference electrode and a carbon fiber traversing the film (*d* = 7 μm) acting as the working electrode. The scan starts at 0 V and sweeps to 1.5 V at 50 mV·s^–1^. In all panels in this figure, the anodic current is represented as positive, in line with IUPAC convention.

Figure 3A shows the voltammetry of 3.5 mM fentanyl in ionic liquid at a glassy carbon disk (*d* = 3 mm) electrode. An anodic peak from fentanyl oxidation can be observed at ∼1.2 V versus platinum wire. Figure S2B shows the voltammetry of fentanyl in bulk ionic liquid at a cylindrical carbon fiber electrode. When the 3.5 mM fentanyl solution was suspended as an ionic liquid film and oxidized at a carbon fiber electrode, a peak is still observed in the voltammetry (Figure 3B); however, there is also limiting current character. This is due to differences in the diffusion profiles (macrodisk versus microcylinder).^15^ Importantly, 3.5 mM fentanyl in the suspended film volume (∼2.4 μL) corresponds to about 3 μg, well below dangerous exposure levels.

To safely mimic the collection of solid aerosols, we introduced fentanyl powder directly into the suspended ionic liquid film by touching the film with a capillary containing a submilligram amount of fentanyl powder (Figure S8). The solid line in Figure 3C shows the voltammetric response when the carbon fiber was moved to the site of powder introduction. One of the limitations of this method is slow molecular diffusion in a viscous ionic liquid. A simple estimation by the Einstein equation
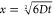
where *x* is the displacement, *D* is the diffusion coefficient, and *t* is the time. This suggests that a diffusing molecule (*D* ∼ 10^–12^ m^2^·s^–1^) introduced near the edge of the suspended film requires days to reach the electrode at the film’s center. In most settings, we expect aerosols to be collected over the surface area, including near to the working electrode (evidenced below with liquid aerosol collection) such that this limitation should not pose an issue for this application. An advantage of the slow diffusion is minimized crosstalk; the species generated at the counter/reference electrode do not influence electrochemistry at the working electrode unless a potential is applied for several hours, even if the loop was miniaturized to be an order of magnitude smaller (0.4 mm). These characteristics can be tuned by changing the suspended ionic liquid.

**Figure 4:**
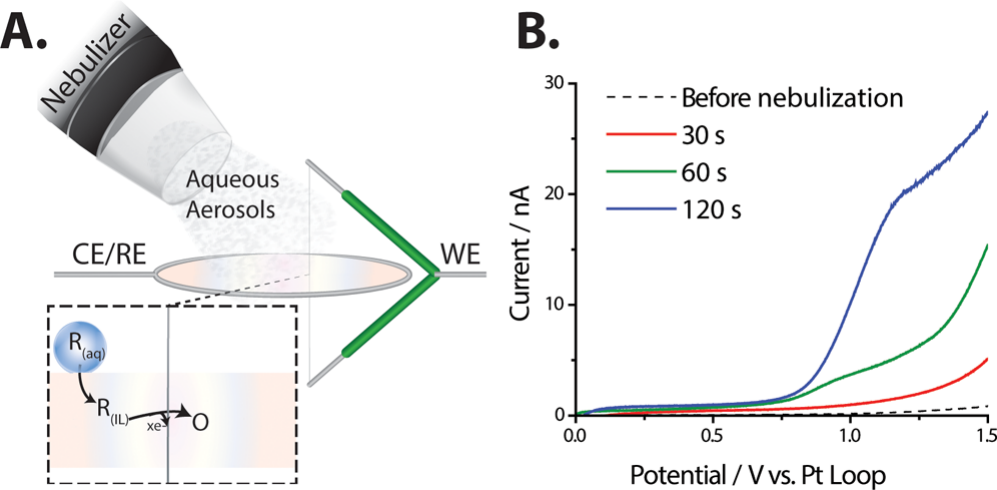
(A) Schematic of liquid aerosol collection experiments. A mesh nebulizer is backfilled with fentanyl-saturated water (>500 μM) and positioned closely to the ionic liquid film. Upon aerosol contact with the film, the fentanyl in the water *R*_(aq)_ partitions into the ionic liquid, *R*_(IL)_, and is oxidized by the biased carbon microwire (WE). (B) Voltammetry of the ionic liquid (1-butyl-3-methylimidazolium hexafluorophosphate) before (dashed line) and after (solid lines) exposure to the fentanyl-containing aerosols. The nebulization was paused, and a voltammetric sweep was collected after subsequent exposures to the nebulized aerosols. The red trace shows the voltammetric sweep after 30 s of exposure to the nebulized aerosols. The green trace shows the voltammetric sweep after a subsequent 60 s of exposure to the nebulized aerosols. The blue trace shows the voltammetric sweep after a subsequent 120 s of exposure to the nebulized aerosols. For all voltammetry, a two-electrode cell was used with a platinum wire loop counter/reference electrode and a carbon microwire (*d* = 7 μm) working electrode. The scan starts at 0 V and sweeps to 1.5 V at 50 mV·s^–1^. In line with the IUPAC convention, the anodic current is represented as positive.

Finally, we employ the suspended electrochemical cell for the collection and detection of fentanyl from liquid aerosols. To controllably introduce aerosols, we positioned a mesh nebulizer ∼1 in. away from the suspended electrochemical cell and nebulized a saturated solution of (>500 μM) fentanyl in water. We note that this experiment was performed in a closed fume hood for safety. Figure 4A shows a scheme of the experimental setup. The dashed line in Figure 4B shows the voltammetric response in a clean suspended film before exposure to the nebulized droplets, where there is low current at 1.2 V. After 30 s, 60 s, and 120 s of subsequent exposures to the nebulized aqueous aerosols, appreciable current is apparent in the voltammetry at 1.2 V, as shown overlaid in Figure 4B. Based on the oxidative onset potential (∼0.7 V), we suggest that the fentanyl can readily partition into the ionic liquid for oxidation. Figure S9 shows that the oxidation of fentanyl in 0.1 M KCl in water occurs at less positive potentials (∼0.5 V). Fentanyl is more soluble in ionic liquid than water, allowing for a liquid/liquid extraction from the aqueous aerosols. We note that the ionic liquid is not totally immiscible with water, and significant swelling of the film occurs with high humidity. This is apparent in the background current in Figure 4B (0–0.5 V). As a control, Figure S10 shows the voltammetric response to the aerosolization of only water, where the recorded currents at 1.2 V are ∼2 nA, an order of magnitude smaller than the current observed with fentanyl oxidation.

The limit of detection is set by the ratio of the faradaic peak current to capacitive current. The capacitive current scales linearly with the effective electrode area and scan rate, whereas the faradaic peak current depends on the diffusive flux profile at the electrode, which is set by the geometry and scale of the film. Additionally, the diffusive flux is slowed by the viscosity of the ionic liquid, and employment of less viscous ionic liquids could improve the signal/noise. Thus, we do not construct a calibration curve to rigorously assess the analytical figures of merit in this initial work. Similar voltammetric techniques for the detection of fentanyl in ionic liquid by direct oxidation on carbon electrodes achieved limits of detection on the order of 10 μM.^8^ For a film 300 μm thick, this would correspond to the detection of <10 ng of fentanyl. Notably, these sensors use pulse techniques to increase sensitivity,^18^ which will be explored in future directions of this work.

The selectivity of this strategy must be understood before a deployable device is useful. This method offers two major pathways for discrimination: (1) the dissolution/partitioning of the analyte into the ionic liquid and (2) the observed peak potentials of the analyte were solubilized by the ionic liquid. Using a hydrophobic ionic liquid helps select highly lipophilic molecules, like fentanyl. Samec previously studied the transfer of a variety of protonated opioids from water to a hydrophobic ionic liquid (tridodecylmethylammonium tetrakis[3,5-bis(trifluoromethyl)phenyl]borate) by voltammetry.^19^ While all opioids tended to accumulate in the ionic liquid, fentanyl and furanyl had the highest partition coefficients. Interestingly, the partition coefficients followed a similar trend as the opioid’s potency. After solubilization, voltammetry offers some selectivity information. In this study, we focused on the appearance of anodic current at 1.2 V for a simple binary metric. Previously, voltammetry of fentanyl in ionic liquid (1-butyl-1-methylpyrrolidinium bis(trifluoromethylsulfonyl)imide), was shown to have multiple characteristic peaks that, considered together, provided discrimination in samples where fentanyl was mixed with common interferents (i.e., glucose, caffeine, acetaminophen, and theophylline).^18^ Future directions will be aimed at understanding the electrochemical signature of fentanyl in hydrophobic ionic liquids and exploring the response of other common drugs and fentanyl analogues in this system.

These sensors show great promise for long-term stability, especially in the context of shelf life, as the ionic liquid effectively does not evaporate. We observed that the suspended ionic liquid film was stable for at least one month. In fact, the film is robust and stays intact after brief contact to surfaces and being dipped into powders/aqueous solutions (Video S1). Like previously reported glove electrochemical cells,^7^ this electrochemical method does not exclude the possibility to sample with a robust liquid film could also avoid accidental aerosolization that could occur as a result of dry sampling. We note that after fentanyl was introduced, it was necessary to reconstruct the electrochemical cell with a clean ionic liquid and a cleaned carbon fiber before taking new measurements. Future work will explore the possibility to construct these electrochemical cells with cost-effective materials toward the development of affordable single-use sensors.

## Conclusions

We have demonstrated the collection and detection of fentanyl captured from powder and sprayed aqueous aerosols by the use of a suspended ionic liquid electrochemical cell. When fentanyl is solubilized by the ionic liquid film, it can be directly oxidized at the carbon fiber working electrode and, without any sample preparation, the current at 1.2 V versus platinum wire reports on exposure levels. The suspended film was designed to have high-surface area:volume for effective aerosol capture and low dilution. Voltammetry in millimolar solutions detects micrograms of fentanyl in the film, an amount well-below the fatal dose (milligram level). We characterized the electrochemical cell with a well-behaved electron mediator using finite element simulations to model the flux and extract the thickness of the film (∼300 μm). While fentanyl detection is of immediate importance, this simple construction can be easily tuned by changing electrode materials, ionic liquid, and dimensions (i.e., scalable), suggesting possible utility for a variety of applications. Notably, the ionic liquid films are readily deployable as they are easy to construct (dipping a loop into ionic liquid), robust (not popping from evaporation or normal use manipulation), and made from a safe and environmentally friendly solvent. Paired with a portable potentiostat, this method provides a simple point-of-care electrochemical device for monitoring fentanyl in aerosols.

## Supporting Information Available

Replicate voltammetry; COMSOL simulation details; experimental setup; voltammetry of fentanyl in bulk ionic liquid solutions; diffusion coefficient calculation; scan rate analysis; and water control for nebulization study (PDF)
Electrochemical cell (MOV)

## Supplementary Material

ac4c01321_si_001.pdf

ac4c01321_si_002.mov
