# Supplementary material for: Capture and Detection of Aerosolized Fentanyl in a Suspended Electrochemical Cell
Source: Anal Chem. 2024 Jun 19;96(26):10648–53. doi: 10.1021/acs.analchem.4c01321 (PMC11223095; doi:10.1021/acs.analchem.4c01321)
Supplement: Supplementary file 1 — ac4c01321_si_001.pdf [file ac4c01321_si_001.pdf]

Capture and Detection of Aerosolized Fentanyl in a Suspended Electrochemical Cell

Kathryn J. Vannoy[a*], Lynn E. Krushinski[a], and Jeffrey E. Dick[a,b*]

[a]Department of Chemistry, Purdue University, West Lafayette, IN, 47907
[b] Elmore Family School of Electrical and Computer Engineering, Purdue University, West Lafayette, IN 47907, USA
*To whom correspondence should be addressed: jdick@purdue.edu

Contents

Voltammetry of ferrocenemethanol and diffusion coefficient calculation …(**Figure S1,** Page S2)

Voltammetry of fentanyl in ionic liquid……………………………………(**Figure S2,** Page S3)

Scheme of the electrochemical cell………………………………………...(**Figure S3,** Page S4)

Scan rate study in suspended ionic liquid film ………………………….(**Figure S4,** Page S5)

Simulation Information…………………………………………(**Figures S5-S6,** Pages S6-S8)

Replicate voltammetry of fentanyl in the suspended film………………..(**Figure S7,** Page S9)

Photographs of introducing powder into the suspended film………………..(**Figure S8,** Page S10)

Voltammetry of fentanyl in an aqueous bulk solution…………………….(**Figure S9,** Page S11)

Nebulized water control…………………………………………………(**Figure S10,** Page S12)

Video of sensor dipped into powder……………………………………………...(**Video S1**)

References…………………………………………………………………………(Page S13)

The diffusion coefficient of ferrocenemethanol was calculated from voltammetry performed in the ionic liquid (1-Butyl-3-methylimidazolium hexafluorophosphate) using the limiting current to an inlaid disk,

$$i_{lim} = 4nFDCr$$

where the limiting current (i_{lim}) is measured to be 24.2 ± 0.9 pA, n is number of electrons (1), F is Faraday's constant (96485 C·mol^{-1}), C is concentration (3.55 mM), and r is electrode radius (5 µm). The diffusion coefficient of ferrocenemethanol in 1-Butyl-3-methylimidazolium hexafluorophosphate was determined to be (3.5 ± 0.1) · 10^{-12} m^2·s^{-1}.

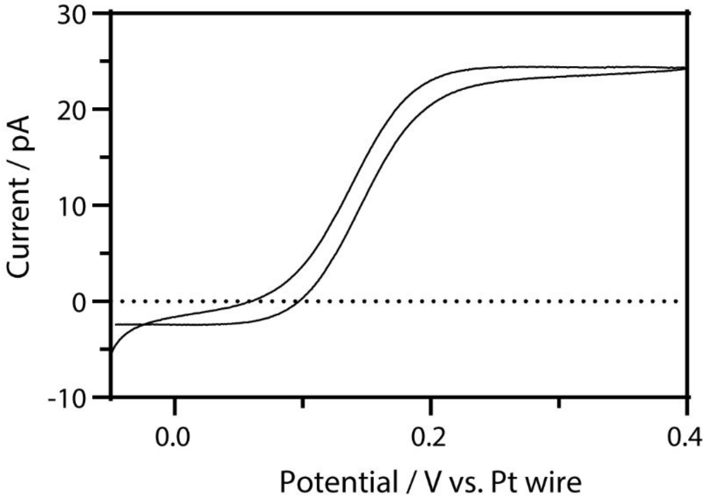

Figure S1. Representative cyclic voltammogram of 3.55 mM ferrocenemethanol in the ionic liquid (1-Butyl-3-methylimidazolium hexafluorophosphate). A two-electrode cell was used with a platinum wire counter/reference electrode and a platinum disk microelectrode (d = 10 µm) working electrode. The scan starts at -0.1 V and sweeps to and from 0.4 V at 1 mV·s^{-1}. In line with IUPAC convention, anodic current is plotted positive.

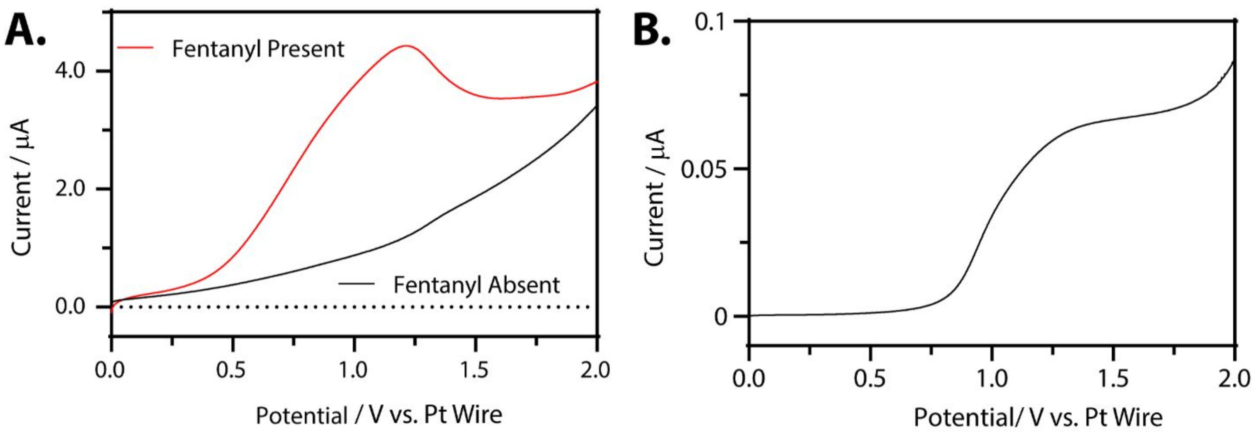

Figure S2. (**A**) Overlaid voltammetry of the ionic liquid (1-Butyl-3-methylimidazolium hexafluorophosphate) with (red) and without (black) ~15 mM fentanyl. A two-electrode cell was used with a platinum wire counter/reference electrode and a glassy carbon disk (*d* = 3 mm) working electrode. The scan starts at 0 V and sweeps to 2 V at 50 mV·s^{-1}. (**B**) Voltammetry of the ionic liquid (1-Butyl-3-methylimidazolium hexafluorophosphate) with ~15 mM fentanyl. A two-electrode cell was used with a platinum wire counter/reference electrode and a carbon fiber (*d* = 7 µm, length unknown) working electrode was dipped into the solution. The scan starts at 0 V and sweeps to 2 V at 50 mV·s^{-1}. Anodic current is plotted positive.

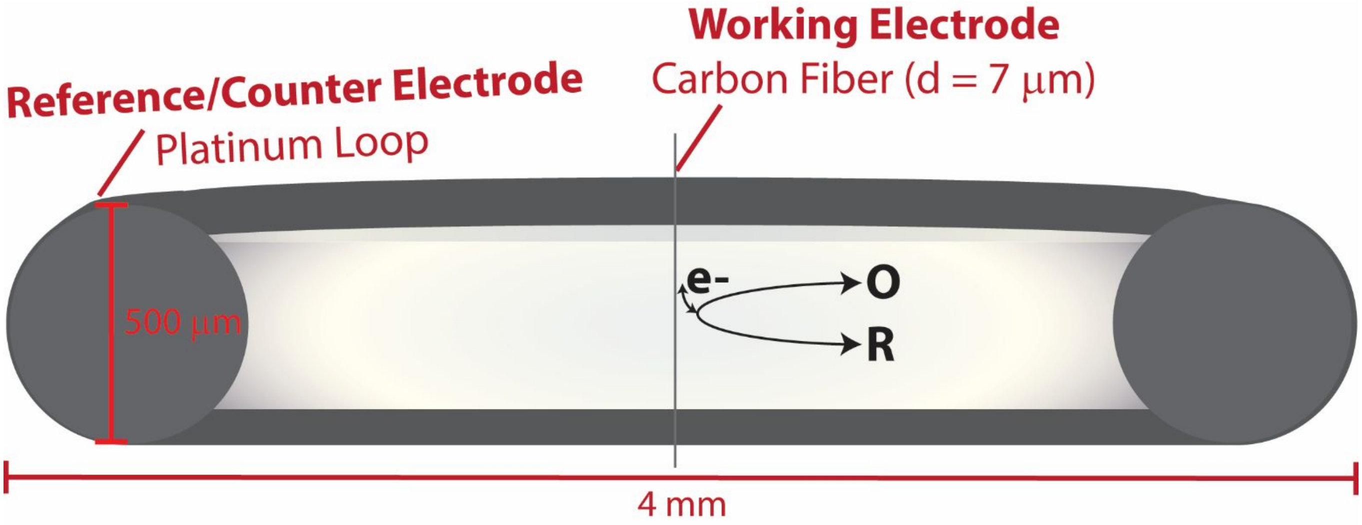

Figure S3. Cross-sectional scheme of the suspended ionic liquid film in a platinum wire (d = 0.5 mm) loop with a carbon fiber (d = 7 μm). A redox couple present in the ionic liquid undergoes oxidation and reduction at the surface of the working electrode.

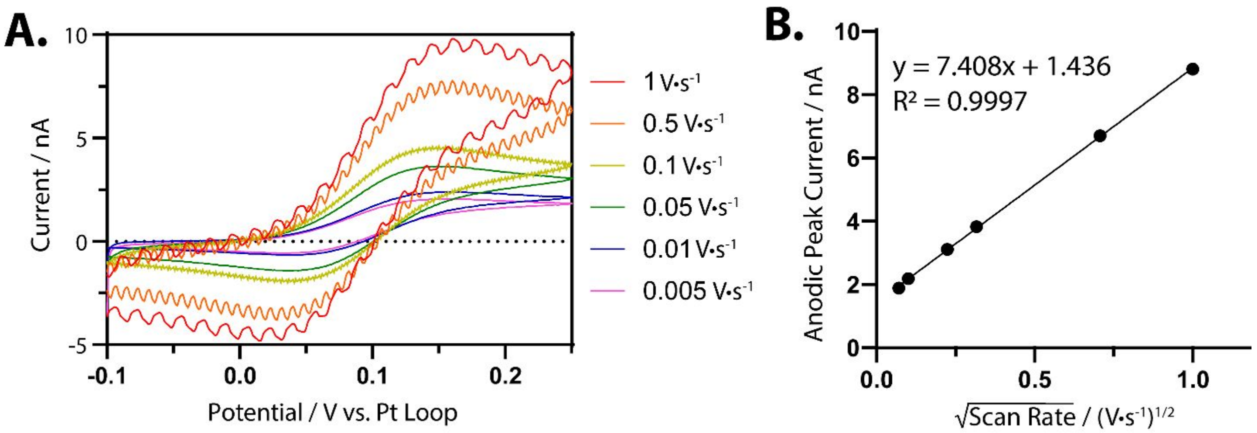

Figure S4. (**A**) Overlay of six cyclic voltammograms collected at scan rates 5 mV·s^{-1} to 1 V·s^{-1}. Voltammetry was performed in a fume hood using the suspended ionic liquid film as a two-electrode cell: the platinum wire loop was used as the counter/reference electrode and a carbon fiber (d = 7 µm) was used as the working electrode. All voltammetry was collected in the same construction: the same ionic liquid film with the same carbon fiber electrode. The scan starts at -0.1 V and sweeps to 0.25 V and the voltammetry was sampled every 0.001 V. (**B**) Plot of the anodic peak currents from panel A *versus* the square rate of scan rate. A linear regression is drawn, and the equation and goodness of fit are inset. In line with IUPAC convention, anodic current is plotted positive in this figure.

Simulation Information

COMSOL Multiphysics 6.0 was used to perform the finite element simulations.

Geometry:

The model was built as 2D axisymmetric and the axis of revolution is the center of the carbon fiber electrode (**Figure S5**). This model assumes that the carbon fiber is perfectly centered in the film and runs perfectly perpendicular, and that the film is a homogenous length across all *r* values (no wetting).

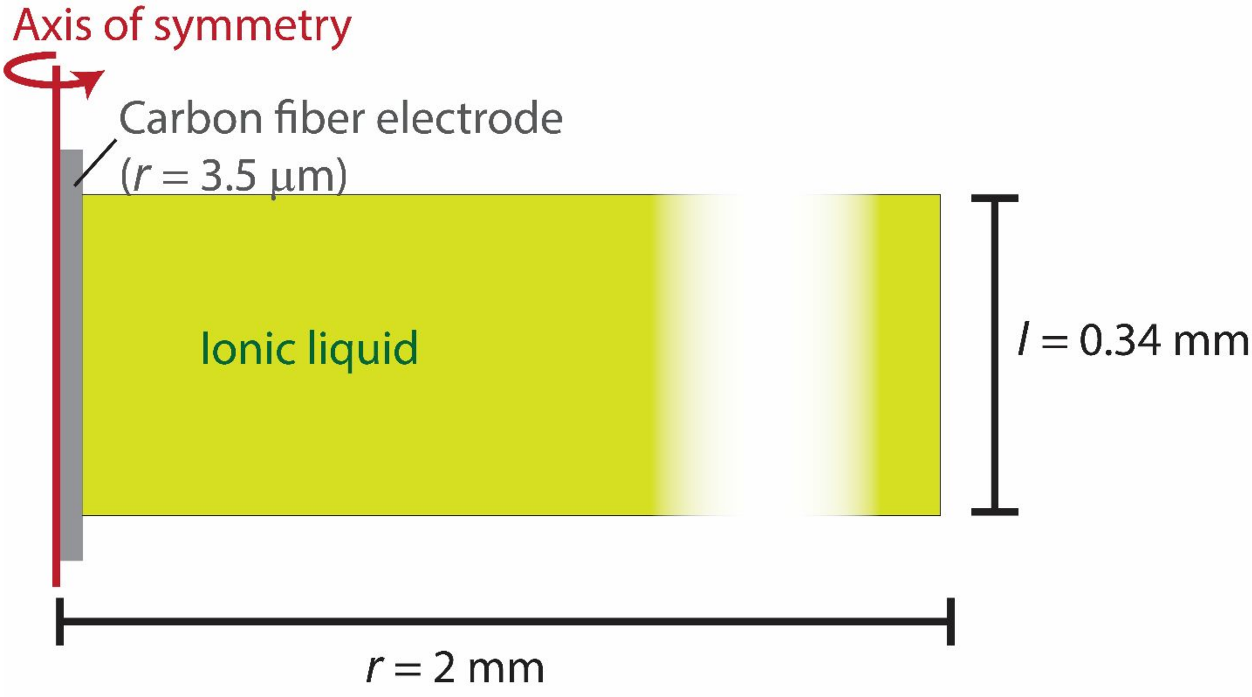

Figure S5. Labeled illustration of 2D axisymmetric geometry used in COMSOL. The scheme is not to scale.

Physics: Transport of Diluted Species

The only mass transport included is diffusion of ferrocenemethanol and ferrocenium methanol in the ionic liquid. A flux boundary condition was set at the interface between ionic liquid and the carbon fiber electrode described by the Butler-Volmer equation

$$\frac{j_{FcMeOH/FcMeOH+}}{F} = \pm\, k^0\left(C_{FcMeOH+}\exp\left(-\alpha\frac{nF}{RT}(E-E^0)\right) - C_{FcMeOH}\exp\left((1-\alpha)\frac{nF}{RT}(E-E^0)\right)\right)$$

where $j_{FcMeOH/FcMeOH+}$ is the flux of FcMeOH/FcMeOH+, k^0 is the heterogenous rate constant, R is the gas constant, T is temperature, α is the transfer coefficient, E is the electrode potential, $E^0$is the formal potential of the FcMeOH/$FcMeOH^+$ couple.

To simulate the change of potential with time a triangular waveform interpolation, where the time component was described by

$$\frac{vertex\ potential - initial\ potential}{scan\ rate}$$

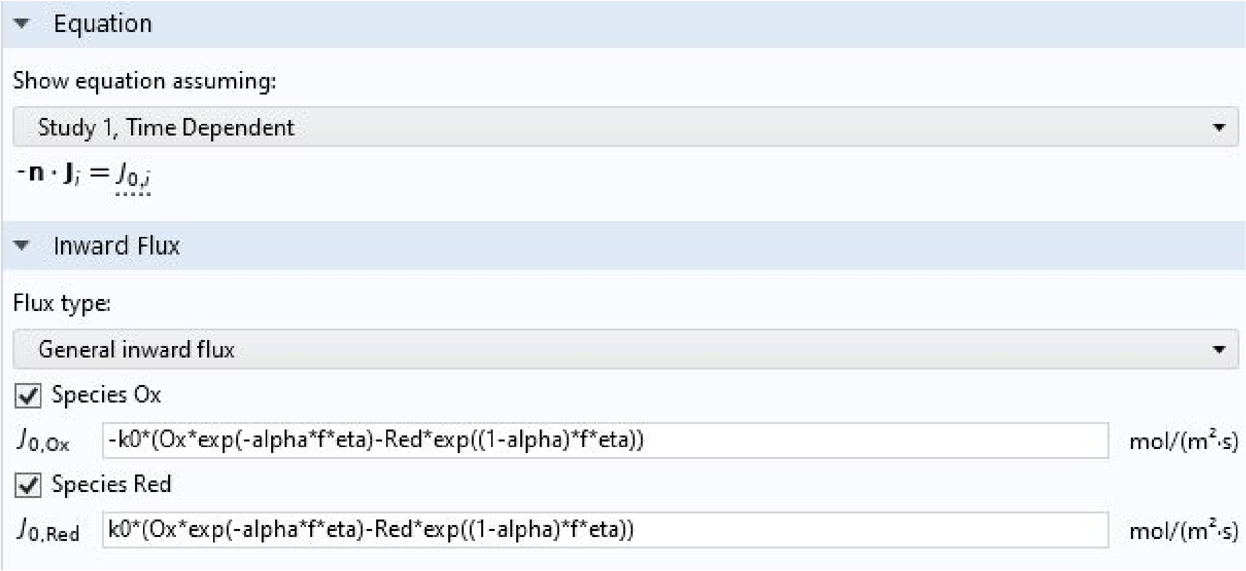

Results:

The current is calculated by line integration of normal diffusive flux of ferrocenemethanol/ferrocenium methanol over the interface between the electrode and the ionic liquid (multiplied by Faraday's constant). The experimental data is input as a table and the simulation is solved varying the height parameter of the ionic liquid/effective electrode. All other parameters are fixed.

Mesh:

A custom mesh was applied to the ionic liquid domain, the boundary of ionic liquid|electrode, and the endpoints of the boundary of ionic liquid|electrode with maximum domain sizes of 10^{-5} m, 10^{-7} m, and 10^{-8} m (**Figure S6**).

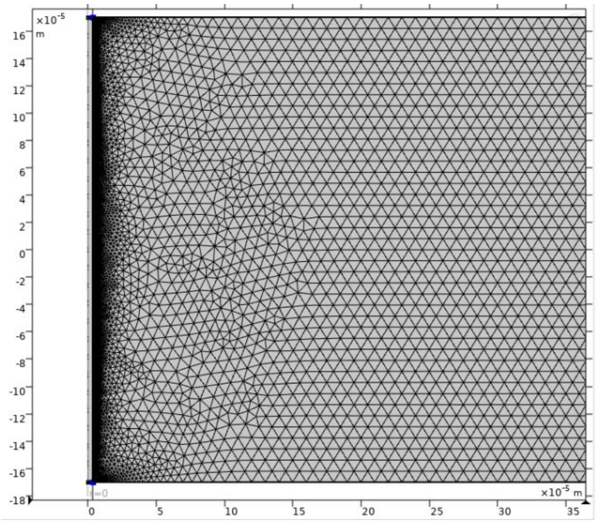

Figure S6. Zoom-in on the electrode|ionic liquid boundary to show meshing.

Parameters:

All parameters were fixed in each simulation except the length on the electrode|ionic liquid, *l.* The diffusion coefficient and formal potential were determined experimentally. The heterogeneous rate constant was used from prior literature.[1]

Parameters

Name	Expression	Value	Description
k0	.00036[cm/s]	3.6E-6 m/s	heterogeneous rate constant
alpha	0.5	0.5	transfer coefficient
Ef	-0.1[V]	-0.1 V	initial/final potential
Ev	0.25 [V]	0.25 V	vertex potential
cRed	3.55 [mM]	3.55 mol/m³	FcMeOH concentration
cOx	0 [mM]	0 mol/m³	FcMeOH+ concentration
v	.05 [V/s]	0.05 V/s	scan rate
ts	abs(Ef-Ev)/v	7 s	time of one sweep
V	0.093 [V]	0.093 V	formal potential
f	F_const/(R_const*T)	39.586 1/V	combination of constants
T	293.15 [K]	293.15 K	temperature
D	3.5e-12 [m^2/s]	3.5E-12 m²/s	diffusion of FcMeOH
l	.34e-3 [m]	3.4E-4 m	length of electrode|ionic liquid
r_e	3.5e-6 [m]	3.5E-6 m	radius of electrode
D_cation	3.5e-12	3.5E-12	diffusion of FcMeOH+

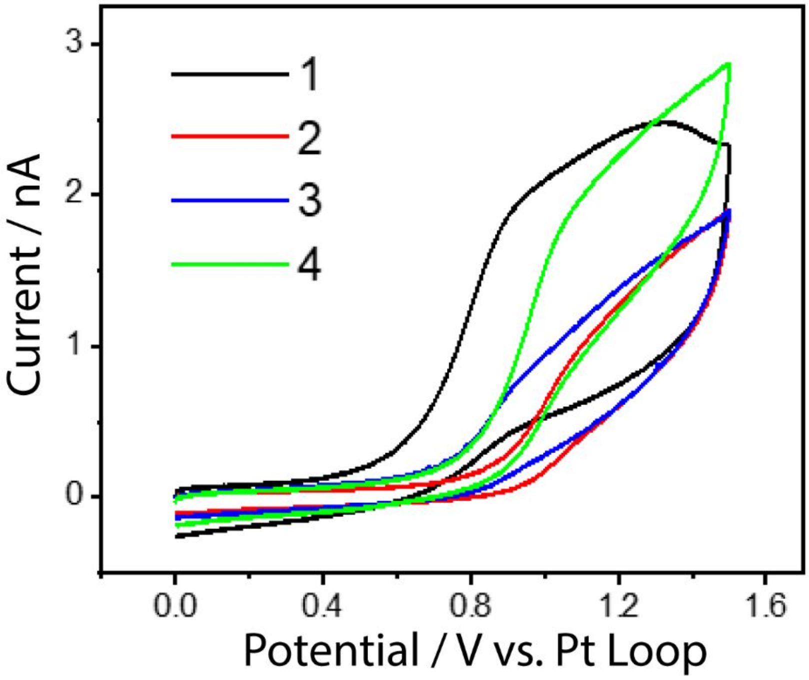

Figure S7. Overlay of four replicates cyclic voltammograms of 1.5 mM fentanyl in the 1-Butyl-3-methylimidazolium hexafluorophosphate (ionic liquid) film. A two-electrode cell was used with a platinum wire loop as the counter/reference electrode and a carbon fiber (d = 7 µm) as the working electrode. Each replicate was collected in different film using a different carbon fiber working electrode. Voltammetry was collected at a scan rate of 50 $mV{\cdot}s^{-1}$ with a from 0 to 1.6 V. In line with IUPAC convention, anodic current is represented as negative.

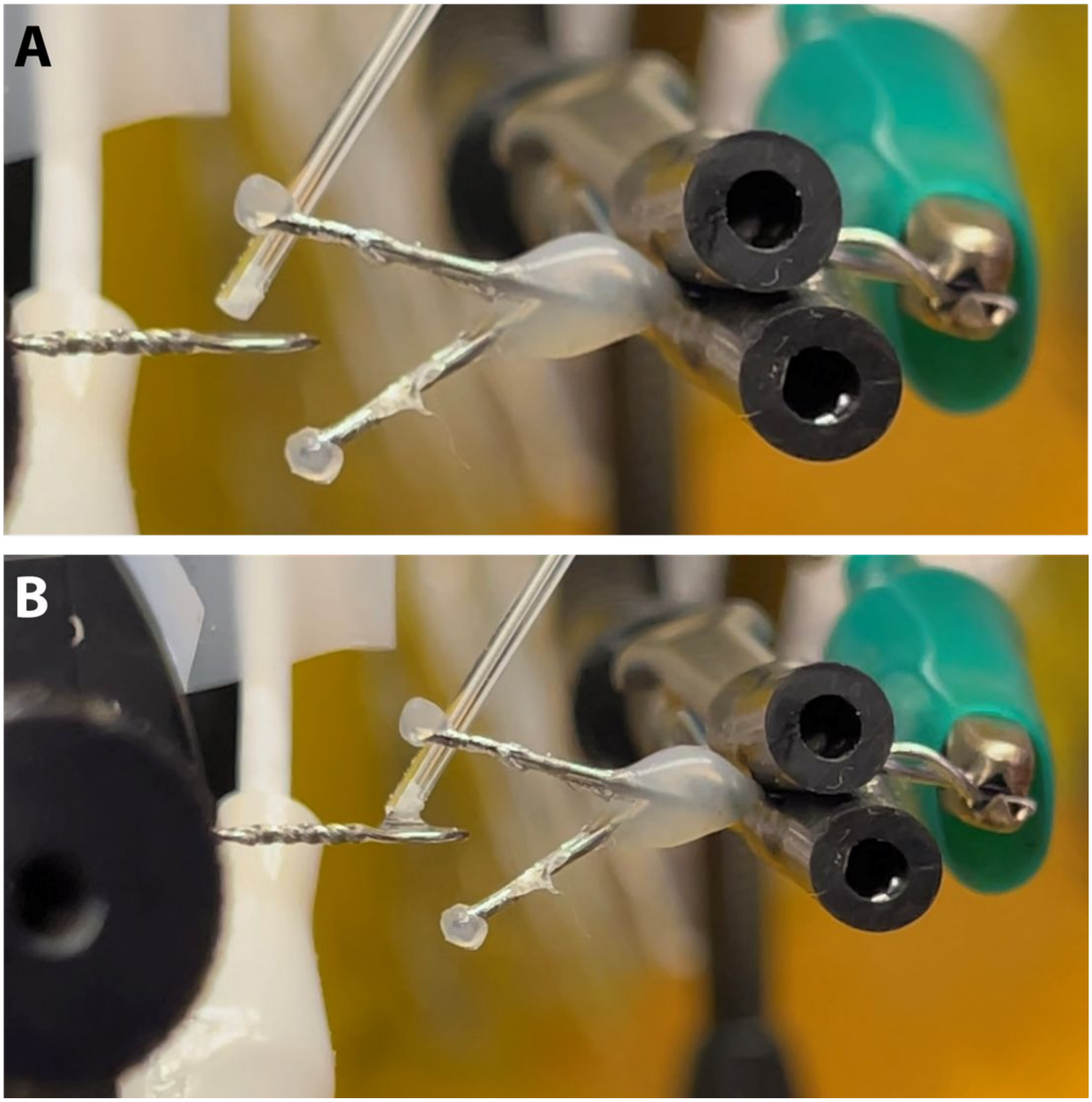

Figure S8. Photographs of introducing fentanyl powder to the suspended ionic liquid film using sub-mg amount of powder in contained in a glass capillary. The capillary filled with fentanyl powder before (**A**) and during (**B**) contact with the ionic liquid bubble.

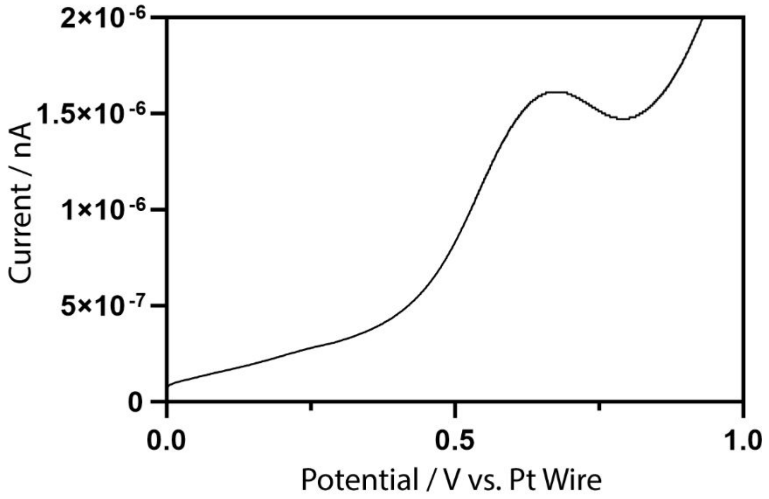

Figure S9. Voltammetric sweep of fentanyl saturated in bulk water with 0.1 M KCl added. A two-electrode cell was used with a platinum wire counter/reference electrode and a glassy carbon disk (*d* = 3 mm) working electrode. The scan starts at 0 V and sweeps to 1 V at 50 mV. In line with IUPAC convention, anodic current is plotted positive.

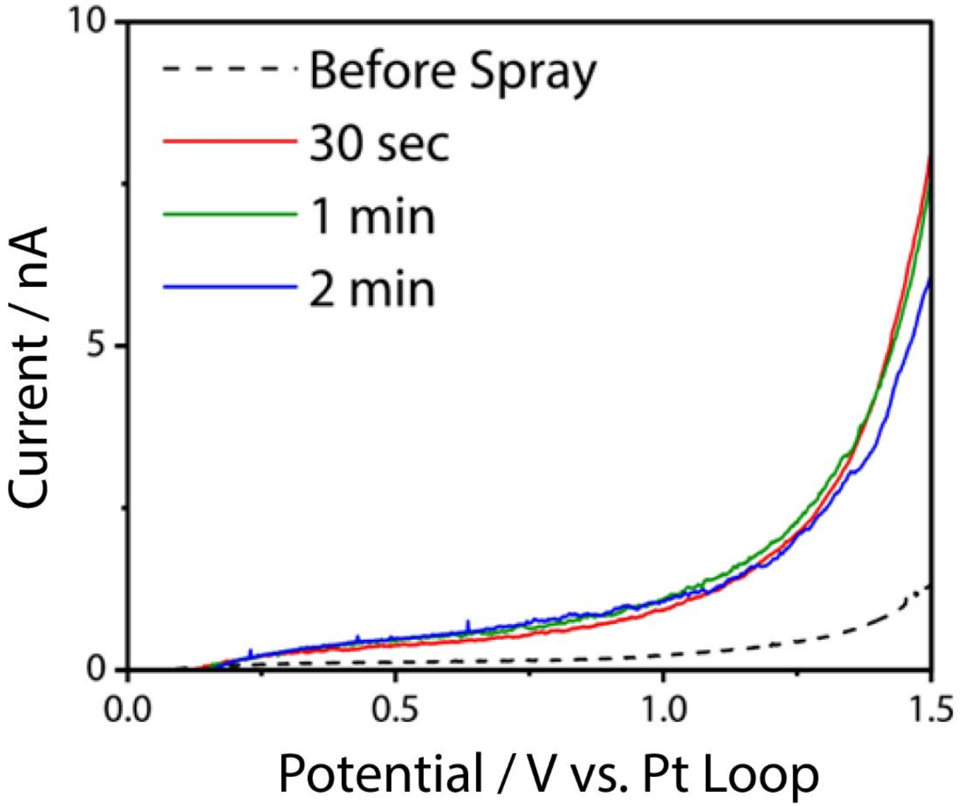

Figure S10. Voltammetry collected before (dashed line) and after the (solid lines) the nebulization of water into the suspended ionic liquid film. Voltammograms were collected with a two-electrode cell: the platinum wire loop was used as the counter/reference electrode and a carbon fiber (d = 7 μm) was used at the working electrode. The scan starts at 0 V and sweeps to 1.5 V at a scan rate of 50 $mV \cdot s^{-1}$.

References

1. Frenzel, N.; Hartley, J.; Frisch, G. Voltammetric and spectroscopic study of ferrocene and hexacyanoferrate and the suitability of their redox couples as internal standards in ionic liquids. *Physical Chemistry Chemical Physics* **2017**, *19* (42), 28841-28852, 10.1039/C7CP05483A. DOI: 10.1039/C7CP05483A.
